# Biomarker Correlates of Survival in Pediatric Patients with Ebola Virus Disease

**DOI:** 10.3201/eid2010.140430

**Published:** 2014-10

**Authors:** Anita K. McElroy, Bobbie R. Erickson, Timothy D. Flietstra, Pierre E. Rollin, Stuart T. Nichol, Jonathan S. Towner, Christina F. Spiropoulou

**Affiliations:** Emory University, Atlanta, Georgia, USA (A.K. McElroy);; Centers for Disease Control and Prevention, Atlanta (A.K. McElroy, B.R. Erickson, T.D. Flietstra, P.E. Rollin, S.T. Nichol, J.S. Towner, C.F. Spiropoulou)

**Keywords:** Ebola, pediatric, endothelium, cytokines, EVD, Ebola virus disease, biomarkers, children, adults, serum analytes, viruses, Ebolavirus, Sudan virus, Uganda, Gulu

## Abstract

Outbreaks of Ebola virus disease (EVD) occur sporadically in Africa and are associated with high case-fatality rates. Historically, children have been less affected than adults. The 2000–2001 Sudan virus–associated EVD outbreak in the Gulu district of Uganda resulted in 55 pediatric and 161 adult laboratory-confirmed cases. We used a series of multiplex assays to measure the concentrations of 55 serum analytes in specimens from patients from that outbreak to identify biomarkers specific to pediatric disease. Pediatric patients who survived had higher levels of the chemokine regulated on activation, normal T-cell expressed and secreted marker and lower levels of plasminogen activator inhibitor 1, soluble intracellular adhesion molecule, and soluble vascular cell adhesion molecule than did pediatric patients who died. Adult patients had similar levels of these analytes regardless of outcome. Our findings suggest that children with EVD may benefit from different treatment regimens than those for adults.

## Abstract 2

Children who had certain endothelial and immune function markers were more likely to survive infection.

Outbreaks of Ebola virus disease (EVD) occur sporadically in sub-Saharan Africa and are associated with exceptionally high case-fatality rates (CFRs). The disease onset is nonspecific and is characterized by abrupt onset of fever, fatigue, headache, myalgia, and gastrointestinal distress 3–13 days after exposure to the virus (*1*). The term hemorrhagic fever has been used to describe this disease process because hemorrhagic manifestations develop in many patients during the course of illness. The *Ebolavirus* genus includes 5 different viruses that result in different CFRs: Ebola virus (EBOV; CFR 57%–90%), Sudan virus (SUDV; CFR 41%–65%), and Bundibugyo virus (CFR 40%) cause fatal infections, but neither Tai Forest virus nor Reston virus has been associated with human fatalities (*2*,*3*).

Pediatric patients have been underrepresented in EVD studies because total numbers of affected children in any given EVD outbreak, whether associated with EBOV, SUDV, or Bundibugyo virus, are usually low because of outbreak dynamics and societal structure. For example, nosocomial EVD infections mostly occur in adults working on hospital wards, and children are not usually caregivers for EVD patients. However, the 2000–2001 SUDV outbreak in the Gulu district of Uganda, the largest recorded EVD outbreak to that point, resulted in 425 cases; 145 cases were in patients <21 years of age, and 55 of these cases were laboratory confirmed (*4*,*5*). The CFR for pediatric patients in this outbreak was lower than for adults (*6*), but the reasons for this increased survival were unknown. The relatively large number of pediatric cases in this outbreak enabled closer investigation of factors associated with increased survival of pediatric patients with EVD.

Samples collected during the Gulu outbreak have been invaluable for advancing understanding of EVD pathophysiology. Studies using these samples found associations between fatal outcomes and elevated liver enzyme levels, renal dysfunction, cytokine dysregulation, and genetic factors (*7*–*9*). Recently, we analyzed serum biomarkers by using samples from the Gulu outbreak and identified associations between cytokines/chemokines, acute-phase reactants, makers of coagulopathy, and markers of endothelial function and patient death, hemorrhage, and viremia (*10*). In this study, we used a series of multiplex assays to measure the concentrations of 55 serum analytes in specimens from patients from the Gulu outbreak to identify biomarkers that had age-specific associations with survival, hemorrhagic manifestations, or both. 

## Materials and Methods

### Study Design

During the 2000–2001 Gulu EVD outbreak, an international response team, including representatives from the US Centers for Disease Control and Prevention (CDC), provided clinical and technical assistance. Serum samples were obtained as part of the management of these patients and were stored in liquid nitrogen. In addition to the samples from the 55 pediatric patients (<21 years of age) who had laboratory-confirmed EVD, we selected samples from 50 adult patients (>21 years of age) who had laboratory-confirmed infection; this selection was designed to be representative of overall sex ratios, hemorrhagic manifestations, and death rates observed during the outbreak. A total of 45 of the 55 pediatric patients (70 specimens total) and 49 of the 50 selected adult patients (127 specimens total) had sufficient serum available for the proposed studies. Data on all patients used in this study, including age, sex, illness outcome, presence of hemorrhagic manifestations, and number of specimens available, are shown in Table 1. 

**Table 1:** Demographics and clinical data for pediatric and adult patients included in analysis of Ebola virus disease outbreak in Uganda, 2000–2001

Patient age, y	Patient sex	Outcome	Hemorrhagic manifestations	No. specimens
1	F	Fatal	N	1
1	F	Fatal	N	1
1	F	Fatal	N	2
1	M	Fatal	N	2
1	M	Fatal	N	1
2	F	Fatal	N	1
2	M	Fatal	N	1
4	F	Fatal	Y	1
6	M	Nonfatal	N	1
7	F	Fatal	Y	1
10	F	Nonfatal	N	1
10	M	Nonfatal	Y	1
10	F	Nonfatal	N	1
11	F	Nonfatal	N	3
12	F	Fatal	N	1
12	F	Nonfatal	N	1
12	F	Fatal	N	3
14	M	Fatal	N	1
14	F	Nonfatal	Y	1
14	F	Fatal	Y	1
15	F	Nonfatal	N	1
15	F	Nonfatal	Y	2
16	F	Nonfatal	N	3
16	M	Nonfatal	Y	1
17	F	Nonfatal	N	6
17	F	Fatal	N	2
17	F	Nonfatal	N	5
18	M	Fatal	N	1
18	M	Nonfatal	Y	1
18	M	Nonfatal	Y	1
18	F	Nonfatal	Y	1
18	M	Nonfatal	Y	1
19	M	Fatal	N	1
19	F	Fatal	Y	1
19	F	Fatal	N	1
20	F	Fatal	N	2
20	F	Fatal	Y	1
20	F	Nonfatal	N	1
20	M	Nonfatal	N	1
20	F	Fatal	N	3
20	M	Fatal	Y	1
20	F	Nonfatal	N	3
21	M	Nonfatal	N	1
21	M	Nonfatal	Y	1
21	M	Nonfatal	Y	2
23	F	Nonfatal	Y	4
23	F	Nonfatal	N	5
24	M	Nonfatal	N	3
25	F	Nonfatal	Y	4
25	F	Fatal	N	2
25	F	Nonfatal	N	6
25	F	Nonfatal	N	1
26	M	Nonfatal	N	1
26	F	Fatal	Y	2
27	M	Fatal	Y	1
27	F	Fatal	N	2
27	F	Fatal	N	1
28	F	Fatal	Y	1
29	F	Nonfatal	N	1
29	F	Fatal	N	2
30	F	Fatal	N	4
30	F	Nonfatal	N	4
30	M	Nonfatal	Y	1
31	F	Fatal	N	1
32	M	Fatal	N	1
32	F	Nonfatal	Y	1
32	F	Fatal	N	3
32	M	Fatal	N	2
34	M	Fatal	Y	1
34	M	Nonfatal	N	3
35	F	Nonfatal	N	6
35	M	Nonfatal	Y	2
35	M	Fatal	N	1
38	F	Fatal	N	4
38	F	Fatal	N	2
40	F	Fatal	Y	1
40	F	Fatal	Y	4
40	F	Fatal	N	2
40	F	Nonfatal	N	4
40	F	Nonfatal	N	4
42	F	Nonfatal	Y	3
42	M	Fatal	N	4
43	M	Fatal	N	5
45	F	Nonfatal	N	1
45	F	Fatal	Y	2
45	F	Nonfatal	N	1
48	M	Nonfatal	N	7
49	M	Fatal	Y	1
50	F	Nonfatal	N	8
58	M	Fatal	N	1
60	F	Nonfatal	Y	1
60	F	Fatal	N	2
60	F	Fatal	Y	2
60	M	Fatal	N	2

Specimens were prioritized for novel analyses first; if a sufficient sample amount was available, serum chemistry analyses were also performed. All samples were inactivated by γ-irradiation (5 × 10^6^ rad) before use, as previously described (*11*). Institutional Review Board approval was obtained before the study was initiated, and an exemption was granted by the CDC Human Research Protection Office.

### Bead-based Multiplex Assays

The following assays were purchased from Affymetrix (Santa Clara, CA, USA) and performed according to the manufacturer’s instructions: a 26-plex assay for granulocyte-macrophage colony-stimulating factor, growth-regulated oncogene α, interferon (IFN) α2, IFNβ, IFNγ, IFNγ-inducible protein 10 (IP-10), interleukin 10 (IL-10), IL-12 (p70), IL-12 (p40), IL-1α, IL-1β, IL-2, IL-4, IL-5, IL-6, IL-8, IL-1 receptor antagonist, monocyte chemoattractant protein 1, macrophage colony-stimulating factor, macrophage inflammatory protein 1α and 1β, soluble CD40 ligand, soluble E-selectin, soluble Fas ligand, tumor necrosis factor α, and vascular endothelial growth factor A; a 2-plex assay for D-dimer and tissue plasminogen activator; a 5-plex assay for plasminogen activator inhibitor 1 (PAI-1), serum amyloid antigen (SAA), regulated on activation, normal T-cell expressed and secreted (RANTES), soluble intracellular adhesion molecule (sICAM) 1, and soluble vascular cell adhesion molecule (sVCAM) 1; and single-plex assays for C-reactive protein and fibrinogen. Single-plex assays for ferritin and cortisol and 2-plex assays for tissue factor (TF) and thrombomodulin were performed according to the manufacturer’s instructions (Millipore, Billerica, MA, USA). For samples with values outside the upper end of the standard curve, additional dilutions were made as necessary to obtain accurate values for all analytes.

### ELISAs

Mannose-binding lectin (Hycult Biotech, Plymouth Meeting, PA, USA) and total IgG (eBioscience, San Diego, CA, USA) ELISAs were performed according to the manufacturers’ instructions. For samples with values outside of the upper range of the standard curve, additional dilutions were made as necessary to obtain accurate values for all analytes.

### Serum Chemistry Testing

A total of 135 of the 197 patient samples had enough volume for serum chemistry analyses. A Piccolo comprehensive metabolic reagent disk was run on the Piccolo xpress Chemistry Analyzer (Abaxis, Union City, CA, USA) to determine serum chemistry values for alanine aminotransferase, albumin, aspartate aminotransferase, alkaline phosphatase, calcium, chloride, creatinine, glucose, potassium, sodium, total bilirubin, total carbon dioxide, total protein, and blood urea nitrogen. 

### Viremia Analysis 

Total RNA was purified from serum samples by using the MagMAX-96 Viral RNA Isolation Kit on the MagMAX Express-96 Magnetic Particle Processor (Ambion, Grand Island, NY, USA). In parallel, RNA was purified from serial dilutions of serum samples from healthy persons that were inoculated with a known titer of SUDV and used to generate a standard curve. Real-time PCR was then performed as previously described (*12*).

### Statistical Analysis

An analysis of variance (ANOVA) was conducted for each of the 55 analytes by using patient sex, patient age, days after symptom onset, viremia, outcome, hemorrhage, and HIV status as independent variables. To represent the normal course of illness, only samples taken 0–15 days after symptom onset were analyzed. All variables were converted to categorical variables because of the number of samples and the distribution of the numerical data. An initial overall α of 0.25 was set, and the Bonferroni inequality was used to arrive at a critical p value of 0.0045 for the individual analytes. Because multiple testing affects the statistical power of the tests, the technique of Yoav and Hochbergs (*13*) was used to control the false discovery rate to balance the power and type 1 error of the analyses. 

**Figure 1:**
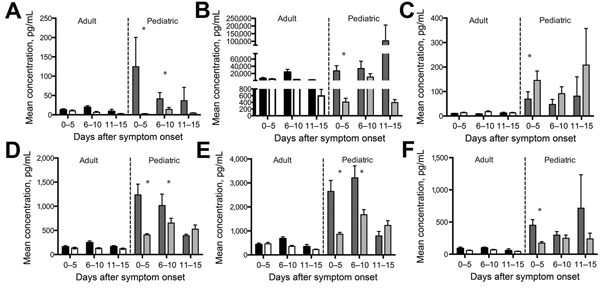
Viral loads for Ebola virus disease patients infected with Sudan virus during outbreak in Uganda, 2000–2001. A) Relative TCID_50_, of pediatric patients (1–21 years of age) compared with those of adult patients (22–60 years of age); B) fatal and nonfatal outcomes for pediatric versus adult patients. Viral load determination was performed on all samples and quantitated by a reverse transcription PCR curve generated from a known titer stock of Sudan virus. TCID_50_, 50% tissue culture infective dose.

**Figure 2:**
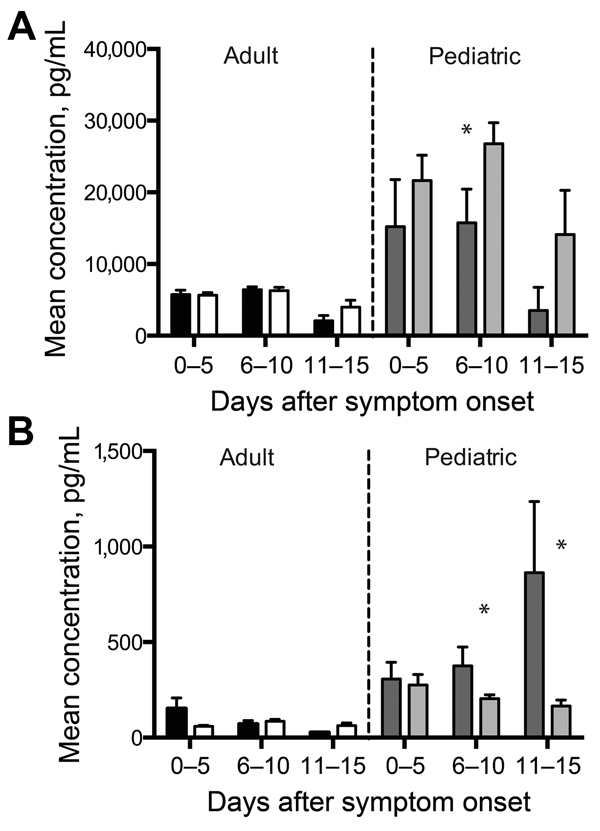
Biomarkers associated with age-dependent survival outcomes for patients with Ebola virus disease: black, adult fatal; white, adult nonfatal; dark gray, pediatric fatal; light gray, pediatric nonfatal. A) Interleukin 10; B) interferon γ–inducible protein 10; C) regulated on activation, normal T cell expressed and secreted; D) soluble intracellular adhesion molecule; E) soluble vascular cell adhesion molecule; F) plasminogen activator inhibitor 1. Mean levels are depicted in each patient group as a function of time after symptom onset. Error bars represent SE; * indicates p<0.05. Numbers of specimens included in each group are as follows: adult fatal at 0–5 days, 27; adult nonfatal at 0–5 days, 20; adult fatal at 6–10 days, 22; adult nonfatal at 6–10 days, 24; adult fatal at 11–15 days, 5; adult nonfatal at 11–15 days, 14; pediatric fatal at 0–5 days, 10; pediatric nonfatal at 0–5 days, 15; pediatric fatal at 6–10 days, 5; pediatric nonfatal at 6–10 days, 13; pediatric fatal at 11–15 days, 2; pediatric nonfatal at 11–15 days, 4.

Using these criteria, 10 analytes showed significant results for age (Table 2). The independent variables were analyzed to measure co-linearity, but no significant variance inflation was observed. After ANOVA, stepwise regression for each significant analyte was used to find the best model, excluding nonsignificant variables (p>0.05). Because a primary focus of this study was the relationship between age and survival in association with these analytes, we added an age/death interaction term for those models where results for age and/or death were significant. This interaction term was significant for 6 analytes (Figure 1). Viremia and death interactions were also examined, but no analytes showed a significant interaction. Age and hemorrhage interactions showed significant results for 2 analytes (Figure 2). We compiled the analytes that were significantly different for these variables and calculated the means and SEs for each time interval. The distribution of the viremia levels was clearly not normal, so the Mann-Whitney U test was used to determine differences in viremia status for adult versus pediatric patients and those with fatal versus nonfatal outcomes within each age group. Patients with no measurable viremia levels were excluded from analysis. A more detailed description of the statistical methods we used is found in our previous study (*10*).

**Table 2:** Characteristics of patients with laboratory-confirmed Ebola virus disease during outbreak in Uganda, 2000–2001*

Characteristic	No. (%) patients	
	Pediatric, n = 37	Adult, n = 49
Sex		
F	23 (62.2)	33 (67.3)
M	14 (37.8)	16 (32.7)
Hemorrhage	15 (40.5)	16 (32.7)
Fatal outcome	14 (37.8)	27 (55.1)

*Pediatric patients were 6–21 y of age, adult patients 22–60 y of age. Eight patients <5 years of age were excluded from this portion of the analysis (see text).

## Results

### Patient Characteristics

Patient characteristics for the final study population are shown in Table 3. CFRs for the 55 laboratory-confirmed pediatric cases varied by age: 76.9% for children <5 years of age (n = 13), 37.5% for children 6–15 years of age (n =16), and 41.6% for adolescents 16–21 years of age (n = 26). In contrast, the CFR for the 161 adults with laboratory-confirmed cases was 56.5%. For the 45 pediatric patients for whom serum samples were available for analysis, the CFR was 100% for children <5 years of age (n = 8), 28.6% for those 6–15 years (n = 14), and 39% for those 16–21 years (n =23). Because all samples were available only from fatal cases for children <5 years of age, we initially excluded these samples from biomarker statistical analyses. However, because this exclusion might have introduced bias, we subsequently repeated the analysis including these 9 additional specimens and found no differences in the statistical significance of the models. Because the CFRs for child and adolescent populations were lower than those for adults, the 3 pediatric age groups were combined to increase the power of the study. 

**Table 3:** Biomarkers that demonstrated an association with age in analysis of Ebola virus disease, Uganda, 2000–2001*

Biomarker	p value
sVCAM	1.72 × 10^−22^
sICAM	3.23 × 10^−22^
SAA	5.42 × 10^−22^
PAI-1	5.77 × 10^−16^
RANTES	1.97 × 10^−10^
MCSF	1.87 × 10^−6^
Total IgG	3.35 × 10^−6^
IP-10	2.34 × 10^−5^
Tissue factor	1.79 × 10^−3^
Interleukin 10	3.20 × 10^−2^

*p values determined by analysis of variance. IP-10, interferon γ–inducible protein 10; MCSF, macrophage colony-stimulating factor; PAI-1, plasminogen activator inhibitor-1; RANTES, regulated on activation, normal T cell expressed and secreted; SAA, serum amyloid antigen; sVCAM, soluble vascular cell adhesion molecule; sICAM, soluble intracellular adhesion molecule..

Patients were considered to have hemorrhagic manifestations if they exhibited any of the following signs: vomiting blood; blood in the stool; or bleeding from the gums, skin, or eyes. We found that a higher percentage of pediatric than adult patients exhibited hemorrhage, but overall CFR remained lower for children than for adults. 

### Viremia

**Figure 3:**
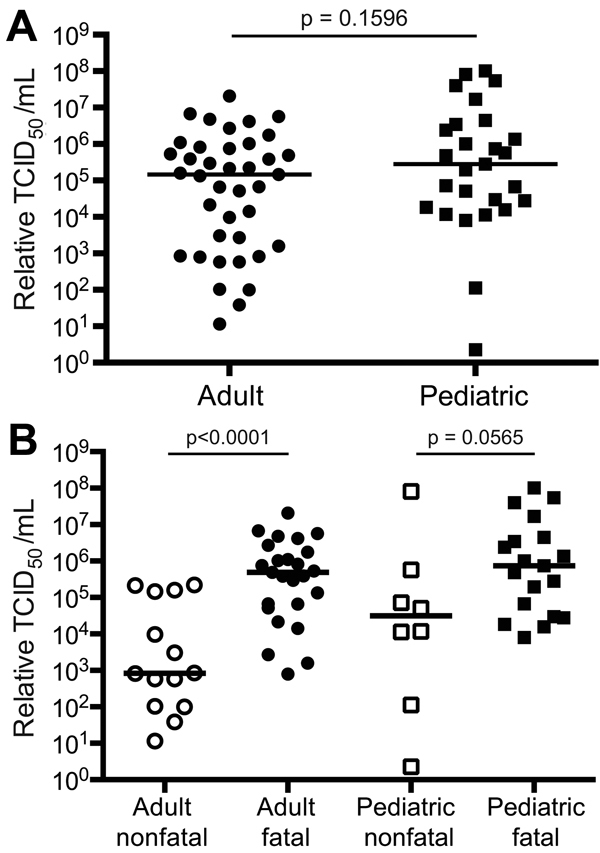
Biomarkers associated with age-dependent hemorrhagic manifestations (heme) for patients with Ebola virus disease: black, adult heme; white, adult nonheme; dark gray, pediatric heme; light gray, pediatric nonheme. A) Serum amyloid antigen; B) plasminogen activator inhibitor 1. Mean levels are depicted in each patient group as a function of time after symptom onset. Error bars represent SE; * indicates p<0.05. Numbers of specimens included in each group are as follows: adult heme at 0–5 days, 9; adult non-heme at 0–5 days, 38; adult heme at 6–10 days, 12; adult non-heme at 6–10 days, 34; adult heme at 11–15 days, 9; adult non-heme at 11–15 days, 10; pediatric heme at 0–5 days, 7; pediatric non-heme at 0–5 days, 18; pediatric heme at 6–10 days, 6; pediatric non-heme at 6–10 days, 12; pediatric heme at 11–15 days, 2; pediatric non-heme at 11–15 days, 4.

To determine whether pediatric patients were more likely to survive as a result of lower levels of viral replication, we measured viremia levels in each sample by using real-time reverse transcription PCR and compared the results with a standard curve generated from stock virus of known titer. No statistically significant differences were found between viral loads in adults and pediatric patients (Figure 3, panel A). Viral loads were higher for patients who died (Figure 3, panel B), as previously demonstrated (*12*); however, in the pediatric population, this difference did not reach statistical significance, likely because of the small sample size and the wide range of observed values in the pediatric patients with nonfatal cases.

### Serum Chemistry Testing

Serum chemistry tests were performed on all samples that had sufficient available volume after initial testing. Blood urea nitrogen, creatinine, and albumin levels varied by age, as expected, given the normal physiological differences between adults and children (data not shown). No age-specific associations were found between any analyte in the serum chemistry results and death or hemorrhage. More labile analytes, such as carbon dioxide and electrolytes, were excluded from analysis.

### Biomarkers of Inflammation

**Figure 4:**
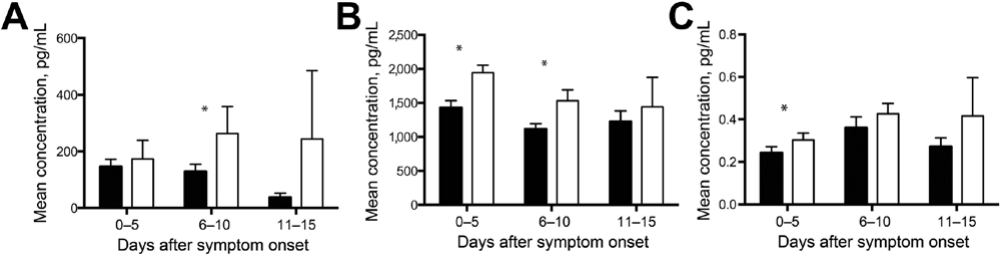
Biomarkers associated with age but not survival outcome or hemorrhage for patients with Ebola virus disease: black, adult; white, pediatric. A) Macrophage colony-stimulating factor; B) total IgG; C) tissue factor. Mean levels are depicted in each patient group as a function of time after symptom onset. Error bars represent SE; * indicates p<0.05. Numbers of specimens included in each group are as follows: adult at 0–5 days, 47; adult at 6–10 days, 46; adult at 11–15 days, 19; pediatric at 0–5 days, 25; pediatric at 6–10 days, 18; pediatric at 11–15 days, 6.

Cytokines and chemokines are a diverse group of proteins that modulate the immune response and have been extensively studied in many different disease processes. We analyzed 25 cytokines and chemokines. Of the 10 analytes that had a statistically significant association with age (Table 2), 4 were cytokines or chemokines, and 3 of those—IL-10, IP-10, and RANTES—were associated with an age-dependent survival outcome (Figure 1, panels A–C). IL-10 and IP-10 levels were higher in pediatric patients who died than in those who survived; adult patients had similar levels of these biomarkers regardless of outcome (Figure 1, panels A, B). Serum samples from this outbreak have been analyzed in the past, and increased levels of IL-10 were reported in patients with fatal outcomes (*8*); however, age was not analyzed in the prior study. RANTES levels were higher in pediatric patients than in adult patients and were further elevated in pediatric patients with nonfatal outcomes (Figure 1, panel C). RANTES is the only biomarker we identified as associated with increased survival in pediatric patients. Macrophage colony-stimulating factor levels were higher in pediatric patients than in adult patients, but no age-specific associations with hemorrhage or survival outcomes were observed (Figure 4, panel A).

The acute-phase response refers to a constellation of host responses that occur during infection and other inflammatory processes (*14*). These responses are classically triggered by proinflammatory cytokines and lead to increased levels of acute-phase reactants. These markers of inflammation are often used clinically to assist in diagnosis and to track a patient’s response to therapy in many infectious or inflammatory processes. Of the acute-phase reactants that we measured—SAA, C-reactive protein, ferritin, and IgG—only SAA and IgG levels demonstrated age-specific associations. SAA levels were higher for pediatric patients than for adult patients and were higher at later times of infection (6–15 days after symptom onset) for pediatric patients without hemorrhagic manifestations than for those with hemorrhagic manifestations (Figure 2, panel A). Total IgG levels were higher for samples from pediatric patients than for those from adult patients (Figure 4, panel B), but no age-specific associations with death or hemorrhage were observed for this biomarker.

### Biomarkers of Endothelial Function

Given the role of the endothelium in maintaining vascular integrity and modulation of hemodynamic stability and the vascular leakage seen in EVD, we included several markers of endothelial function in our study (sICAM, sVCAM, and soluble E-selectin). sICAM and sVCAM demonstrated an age-specific association. ICAM and VCAM are expressed on endothelial cells and upregulated in response to proinflammatory cytokines. Both factors are shed from the surface of activated endothelial cells and can be measured in their soluble form in the serum (*15*). At 0–10 day after symptom onset, pediatric patients had higher levels of sICAM and sVCAM than did adults, and pediatric patients who died had higher levels of both factors than did those who survived (Figure 1, panels D, E).

### Biomarkers of Coagulopathy

The frequent presence of hemorrhagic manifestations in EVD patients and the increased frequency seen in our pediatric population warranted an examination of the measureable factors that control coagulation and fibrinolysis. We measured PAI-1, fibrinogen, tissue plasminogen activator, D-dimer, thrombomodulin, and TF in all patient samples. PAI-1 levels were elevated in pediatric patients, and more so in those who died and those who had hemorrhagic manifestations (Figure 1, panel F; Figure 2, panel B). TF levels were slightly elevated in pediatric patients (Figure 4, panel C), but no age-specific associations with hemorrhage or death were shown.

## Discussion

Differences in disease severity for patients of different ages are not uncommon in infectious diseases. For example, tuberculosis is associated with disseminated disease in children <5 years of age and focal pulmonary disease in adults but causes infrequent and mild disease in school-aged children and adolescents (*16*). A similar pattern was observed in this evaluation of patients infected with SUDV. Two possible explanations for the increased rate of death among children <5 years of age are the contributions of co-occurring conditions or the immature immune systems in children of this age. We extensively assessed biomarkers in serum samples from this SUDV outbreak and found that IL-10, IP-10, RANTES, sICAM, sVCAM, and PAI-1 were higher in pediatric patients than in adult patients and were also associated with specific outcomes in pediatric patients. 

RANTES was the only factor we studied that demonstrated an association with higher survival rates in children. A known chemoattractant for monocytes and T cells, RANTES is produced by many cell types, including endothelial cells and macrophages (*17*), and plays a role in activation and proliferation of antigen-specific T cells (*18*). Decreased levels of RANTES in adult patients infected with chikungunya virus and in children infected with respiratory syncytial virus have been associated with more severe disease (*19*,*20*), and lower RANTES levels in children have been associated with death from cerebral malaria (*21*). RANTES^−/−^ mice infected with lymphocytic choriomeningitis virus show decreased CD8+ T cell cytokine production and cytotoxic ability coincident with higher viral loads (*22*). Lymphocyte apoptosis has been seen in vitro in response to EBOV infection (*23*), and SUDV patients who died have had lower numbers of T cells, CD8+ T cells, and activated CD8+ T cells (*24*). CD8+ T cells were critical for EBOV survival in mouse models (*25*,*26*) and a nonhuman primate model (*27*). Therefore, it is not surprising that higher levels of RANTES were associated with survival in our study. The data from all of these studies suggest that, during SUDV infection, RANTES could recruit and activate T cells, leading to a stronger SUDV-specific T cell–mediated response and thus to improved survival. The occurrence of this phenomenon only in children is notable, but in models of familial hypercholesterolemia, the monoctytes of children, but not of adults, have increased RANTES expression (*28*), which suggests that children might have a greater capacity for RANTES production than do adults.

IL-10 levels were significantly elevated at the earliest times of infection in pediatric patients who died. The role of IL-10 in inhibiting antigen-stimulated T cell proliferation (*29*) supports the assumption that a T cell–mediated response is critical for survival during EVD. Levels of sICAM and sVCAM for children are normally higher than for adults (*30*), and the levels that we detected in surviving pediatric patients were consistent with these normal levels. Pediatric patients who died had sICAM and sVCAM levels 2–3 times above the reference range 0–10 days after symptom onset, but these levels dropped to within the reference range at 11–15 days. This pattern may reflect early excessive, and ultimately detrimental, endothelial activation in these patients. Consistent with this theory are the increased PAI-1 levels also seen in pediatric patients who died; PAI-1 is released by endothelial cells in response to activating cytokines (*31*).

IgG levels were higher in samples from pediatric patients than in samples from adult patients; this difference is notable because children usually have slightly lower levels of total IgG than do adults (*32*). The higher IgG levels might suggest a higher degree of immune activation, perhaps secondary to other infectious co-existing conditions, which are likely to be present in children living in a rural area of Africa. Consistent with this theory, high levels of malarial parasitemia have been associated with higher total levels of IgG in children in The Gambia (*33*).

We also observed associations between age and hemorrhagic manifestations for PAI-1 and SAA levels. Elevated PAI-1 levels in pediatric patients with hemorrhagic manifestations likely represent the overactive endothelium and not a functional inhibition of fibrinolysis, since PAI-1 activity is likely to be low, as it rapidly converts to the inactive form under physiologic conditions (*34*). Normal levels of SAA in children and adults are 10–90 ng/mL, and much higher levels can be seen in disease states (*35*). In our study, higher SAA levels were seen in pediatric patients without hemorrhagic manifestations than in those with hemorrhagic manifestations. These levels were also higher than those seen in adults, regardless of the presence or absence of hemorrhagic manifestations. SAA is known to induce TF production by monocytes (*36*); we found that levels of TF were higher in pediatric patients , but these levels were not outside the normal range for adults or children and were not associated with hemorrhage or its absence. SAA treatment of endothelial cells causes decreased production of nitric oxide (NO) synthase and decreased NO bioavailability (*37*); elevated SAA levels in pediatric patients could have been associated with decreased levels of NO, and, therefore, with decreased vasodilatation and hemorrhage. However, the juvenile endothelium has decreased responsiveness to NO (*38*) and increased levels of circulating asymmetric dimethylarginine, an inhibitor of NO synthase (*39*); thus, altered levels of NO might not have functional consequences in pediatric patients. Alternatively, another, as yet undefined, function of SAA could be responsible for its association with pediatric patients who did not experience hemorrhagic manifestations. Finally, despite the association of PAI-1 with both hemorrhage and death, no statistically significant differences in survival rate were observed between patients with without hemorrhagic manifestations (p = 0.23). This finding suggests that these physiologic observations about hemorrhage are not causally related to survival.

An overactive endothelial response, as evidenced by elevated sICAM, sVCAM, and PAI-1 levels, was associated with death in children and adolescents. However, the adults in our study did not seem to be affected by this phenomenon. Co-existing conditions in the pediatric patients, such malaria or other childhood illnesses, could have contributed to endothelium reactivity, or this finding could be secondary to the known physiologic differences that exist between the adult and juvenile endothelium. Underscoring the importance of these age-specific endothelial differences, clinical trials using drugs that target these differences in treatment of cerebral malaria in children are underway (*40*).

In summary, our data suggest that different pathophysiologic mechanisms of disease may be at work in pediatric patients, and children may benefit from different treatment than their adult counterparts. Therapeutic interventions targeted at decreasing endothelial activation in pediatric patients early during the course of infection might include drugs that affect endothelial activation, such as statins. The clear association between survival and increased RANTES in pediatric patients also suggests that a better understanding of the mechanisms and molecular consequences of increased levels of this chemokine could be useful in future therapeutic design, especially in design of drugs that induce a stronger, earlier, antigen-specific T cell response.
